# Intrinsic determinants of prion protein neurotoxicity in *Drosophila*: from sequence to (dys)function

**DOI:** 10.3389/fnmol.2023.1231079

**Published:** 2023-08-14

**Authors:** Alessandro Cembran, Pedro Fernandez-Funez

**Affiliations:** ^1^Department of Chemistry and Biochemistry, University of Minnesota Duluth, Duluth, MN, United States; ^2^Department of Biomedical Sciences, University of Minnesota Medical School, Duluth, MN, United States

**Keywords:** molecular dynamics, prion protein, protein structure, structure-function, *Drosophila*

## Abstract

Prion diseases are fatal brain disorders characterized by deposition of insoluble isoforms of the prion protein (PrP). The normal and pathogenic structures of PrP are relatively well known after decades of studies. Yet our current understanding of the intrinsic determinants regulating PrP misfolding are largely missing. A 3D subdomain of PrP comprising the β2-α2 loop and helix 3 contains high sequence and structural variability among animals and has been proposed as a key domain regulating PrP misfolding. We combined *in vivo* work in *Drosophila* with molecular dynamics (MD) simulations, which provide additional insight to assess the impact of candidate substitutions in PrP from conformational dynamics. MD simulations revealed that in human PrP WT the β2-α2 loop explores multiple β-turn conformations, whereas the Y225A (rabbit PrP-like) substitution strongly favors a 3_10_-turn conformation, a short right-handed helix. This shift in conformational diversity correlates with lower neurotoxicity in flies. We have identified additional conformational features and candidate amino acids regulating the high toxicity of human PrP and propose a new strategy for testing candidate modifiers first in MD simulations followed by functional experiments in flies. In this review we expand on these new results to provide additional insight into the structural and functional biology of PrP through the prism of the conformational dynamics of a 3D domain in the C-terminus. We propose that the conformational dynamics of this domain is a sensitive measure of the propensity of PrP to misfold and cause toxicity. This provides renewed opportunities to identify the intrinsic determinants of PrP misfolding through the contribution of key amino acids to different conformational states by MD simulations followed by experimental validation in transgenic flies.

## Introduction

Prion diseases are a heterogeneous group of degenerative brain disorders (Mathiason, 2017; Scheckel and Aguzzi, 2018) that present with symptoms overlapping other neurological disorders but are distinguished by their aggressive progression and fatal outcomes. Intriguingly, these conditions have pathological and molecular counterparts in several mammals, including scrapie in sheep and goats, chronic wasting disease (CWD) in cervids, and several other diseases caused by the experimental or accidental transmission of prions to cattle, rodents, felines, mustelids, and others (Chandler and Fisher, 1963; Zlotnik and Rennie, 1963, 1965; Chandler, 1971; Wells et al., 1987; Wilesmith, 1988; Winter et al., 1989; Kirkwood and Cunningham, 1994; Sigurdson and Miller, 2003). Prion diseases are quite exceptional because a single agent – a protein – is responsible for sporadic, genetic, and infectious manifestations of the disease. Remarkably, the agent responsible for these conditions is a small protein, the prion protein (PrP). More specifically a misfolded conformation of PrP that aggregates into highly stable assemblies, spreads from cell-to-cell, and causes aggressive neuronal loss that results in spongiform degeneration of the brain. It is thus remarkable that a simple protein can be responsible for multiple clinical entities, disease inheritance, and disease transmission, representing a unique situation in the animal world (Prusiner, 1998; Colby and Prusiner, 2011; Kraus et al., 2013). Hence, PrP is a strange and fascinating protein that has been under investigation for 40 years, which has resulted in extensive resources for conducting biochemical, biophysical, and computational experiments.

**Figure 1:**
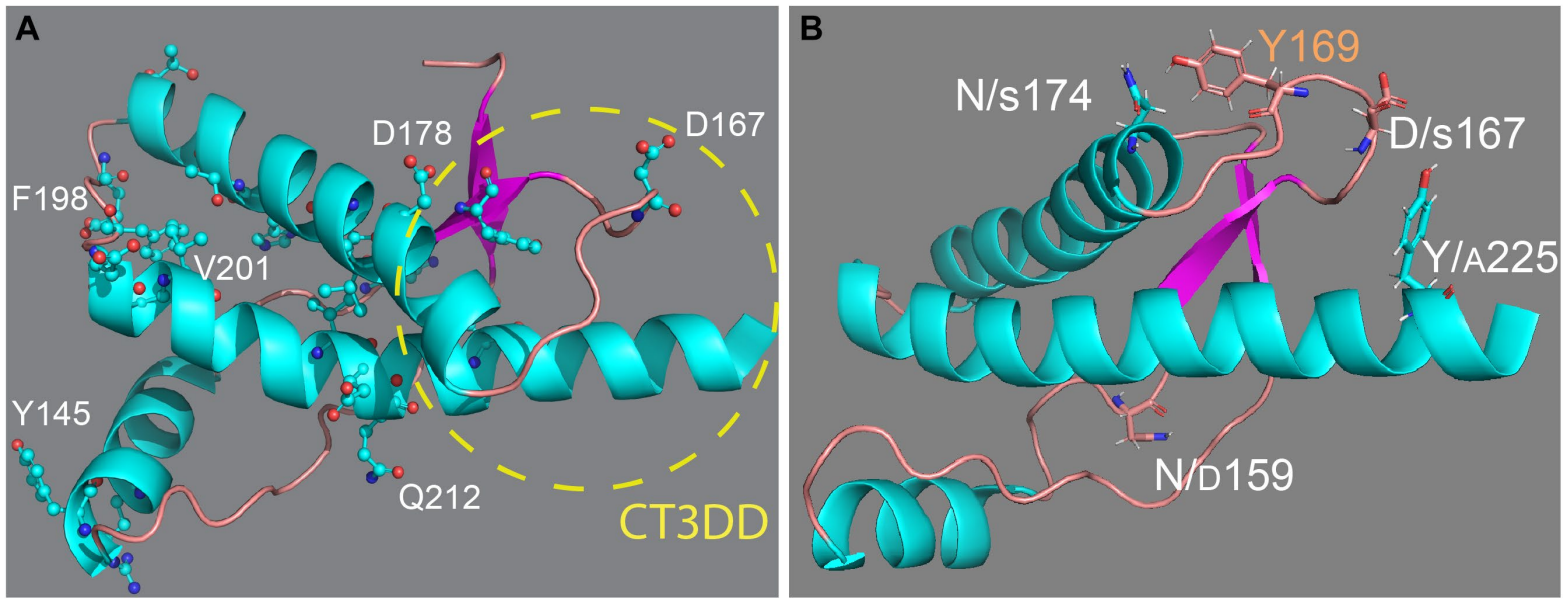
Structure of the globular domain of human PrP. **(A)** Distribution of key pathogenic mutations and the CT3DD. Only a few are identified in different subdomains. **(B)** Distribution of outstanding substitutions in animals resistant to prion disease. The large letters correspond to human PrP and the smaller correspond to different animals. Y169 is a highly conserved residue with a key role in the dynamics of the CT3DD. Created with PyMOL.

PrP is a relatively simple, 230 amino acids-long (after maturation) glycoprotein attached to the extracellular membrane by a C-terminal glycosylphosphatidylinositol (GPI) anchor. It contains an unstructured N-terminal domain with five octarepeats and a small globular C-terminus domain with three α-helices and a short β-sheet. This structure is highly conserved among mammals, suggesting evolutionary constraints for an important physiological function, which has only recently started to come into focus (Wulf et al., 2017; Watts et al., 2018; Panes et al., 2021; Kovac and Curin Serbec, 2022). Classic studies identified a key role for a 3D domain in the C-terminal region consisting of the β2-α2 loop and distal helix 3, termed here the C-terminal 3D domain (CT3DD) (Figure 1A). Remarkably, this is a region of high sequence variability, the proposed binding site of a hypothetical protein (Protein-X) necessary for PrP conversion, and the most prominent surface interaction site by structural studies (Telling et al., 1995; Billeter et al., 1997; Kaneko et al., 1997). It is well established that misfolded PrP conformations are the causative agents of prion diseases (Prusiner, 1998). PrP is known to misfold and assemble into different aggregates, including oligomers, protofibers, and fibers that may play different roles in disease pathogenesis and transmission. The transmissible agent, known as PrP^Sc^ (scrapie PrP) or PrP^res^ (resistant PrP), contains PrP and other molecules and is highly resistant to denaturing agents and proteases (Prusiner, 1998). Other PrP assemblies distinct from PrP^Sc^ are thought to contribute to neurodegeneration, including PrP^Sc^ intermediates, toxic PrP oligomers (PrP^*^), soluble PrP lethal (PrP^L^), transmembrane topologies, and cytosolic PrP (Hegde et al., 1998; Ma et al., 2002; Hegde and Rane, 2003; Harris and True, 2006; Chiesa et al., 2008; Gambetti et al., 2008; Mercer and Harris, 2023). Biophysical analyses indicate that the structural changes during PrP misfolding involve a loss of helical content in the globular domain, from 42 to 30%, and an increase in β-sheet content, from 3% to over 40% (Pan et al., 1993; Perez et al., 2010; Christen et al., 2013). Understanding the rules governing the conformational dynamics of PrP is critical to understand disease risk and, eventually, develop therapeutic agents that can inhibit PrP misfolding and disease. This paper will focus on the impact of sequence variation on PrP conformational dynamics and toxicity. Extensive structural information is available for PrP from different animals and for human pathogenic mutants. Still, decades of structural studies have not yet identified the rules governing PrP dynamics, misfolding, toxicity, and disease susceptibility.

### Structure of PrP globular domain

The unique nature of PrP as a transmissible agent causative of devastating brain disorders led to significant interest to reveal its structural features, resulting in the determination of many PrP structures. This is a vast resource for comparative studies that is unmatched by other proteins. The nuclear magnetic resonance (NMR) structure for PrP in solution was resolved in the late 1990’s, before the X-ray structure (Riek et al., 1996, 1997; James et al., 1997; Liu et al., 1999; Knaus et al., 2001). NMR can be done with proteins in solution, resolving one of the limiting steps in crystallography, which requires the crystallization of highly purified molecules. Full-length and C-terminal domain PrP from both mouse and Syrian hamster were characterized first and the subsequent determination of human, sheep, and bovine PrP confirmed the high conservation of the globular domain (Zahn et al., 2000; Knaus et al., 2001). NMR better captures conformational dynamics, with the ability to reveal areas of increased structural variability in PrP. For instance, elk, deer, bank vole, horse, and wallaby PrP display a prominent 3_10_-turn in the β2-α2 loop (also known as rigid loop), whereas in the rest of PrPs including human, a β-turn is the predominant conformation (Lysek et al., 2005; Perez et al., 2010). Unlike NMR, in X-ray structures the β2-α2 loop always acquires a 3_10_-turn, indicating a bias for the best structure capable of crystallization. The conformational heterogeneity of this loop provides opportunities to investigate its sequence determinants and the consequences of manipulating this key domain within the globular domain.

Despite the small size and relative simplicity of PrP, the molecular mechanisms mediating PrP misfolding remain to be elucidated. New mechanistic knowledge can be gained when protein function can be paired with sequence variation. Significant variation in PrP sequence comes from two main sources: natural variation in mammals and mutations causative of human inherited prion diseases. Unfortunately, sequence variation alone does not provide sufficient mechanistic information despite the ability to accurately model secondary and tertiary protein structures. Most natural sequence variations in PrP do not disrupt the conserved structure of the globular domain; thus, understanding the impact of these changes requires sophisticated analyses of the local and global dynamics of the globular domain.

### Pathogenic mutations in inherited prion diseases

In humans, more than 50 mutations, most of them missense mutations, result in at least three different inherited clinical presentations: Creutzfeldt-Jakob disease (CJD), Gerstmann-Straussler-Scheinker (GSS) disease, and fatal insomnia (FI) (Appleby et al., 2022) 33 missense mutations map in the globular domain vs. only 7 in the flexible domain despite their comparable length (110 vs. 100 residues, respectively) (Figure 1A). Note that throughout the paper we identify amino acid position based on the human PrP sequence to avoid confusion with PrP from other species. This uneven distribution of pathogenic mutations supports the central role of the globular domain in PrP misfolding and disease. The dominant inheritance of PrP mutations suggests gain-of-function mechanisms in which the mutant allele acquires a novel function likely due to changes in its biogenesis or folding dynamics. Whereas nonsense mutations leading to truncated PrP and artificial mutations can alter the basic PrP biogenesis and / or stability (Hegde et al., 1998; Ma et al., 2002; Sanchez-Garcia et al., 2013; Ning et al., 2014), pathogenic missense mutants are more likely to introduce subtle local effects that disrupt its conformational dynamics, i.e., making PrP *more likely* to misfold. In fact, most mutations accumulate in helices 2 and 3, two key structural elements of the globular domain (Lloyd et al., 2011). The loop connecting α2-α3 has only 5 residues, but 2 of them accumulate missense mutations, which agrees with the importance of this loop in modulating the flexibility of the two helices and their interaction with helix 1. In contrast, the α2-β2 loop has 8 residues and accumulates only 1 missense mutation, perhaps revealing a low tolerance to conformational disruptions. Many pathogenic mutations are conservative in nature, i.e., V → I at 180, 203, and 210, which are proposed to cause steric strains within the hydrophobic domain due to the larger size of Iso. Since these mutants cause dominant disease inheritance, they are likely to introduce significant alterations in the dynamics of the globular domain. Overall, pathogenic mutations are expected to increase the mobility of helices 1 and 2 and disrupt their interface with other key domains, namely helix 1 and the α2-β2 loop (Biljan et al., 2017; Rossetti and Carloni, 2017).

### The PrP zoo: clues from susceptible and resistant animals

Prion diseases affect humans and other mammals, but not all mammals are equally susceptible to prion diseases. Only a small group of ungulates are afflicted by endemic prion diseases, including scrapie in sheep and goats and chronic wasting disease in deer, moose, and elk. The history of prion diseases in animals is marked by two landmark events. In the 1960’s human prion diseases were classified as infectious diseases, leading to transmission studies in animals. Some animals developed a similar disease (apes, monkeys, cats, rats, and mice) while others proved resistant (rabbits) (Gibbs and Gajdusek, 1973; Barlow and Rennie, 1976). The second big episode was the mad cow outbreak of the 1980’s in which many farm and zoo animals were exposed to prions from infected sheep. New groups of animals showed susceptibility to prion diseases, including felines and mustelids, whereas horse, dog and other canids, and pig were notable for their resistance (Kirkwood and Cunningham, 1994). The mechanisms underlying this intrinsic variability in susceptibility to prion disease can be exploited to dissect the rules governing PrP dynamics and conversion to pathogenic conformations.

Persuasive evidence indicates that the different animal susceptibility to prion diseases is mediated by intrinsic factors, i.e., the PrP sequence regulating the structure and dynamics of the globular domain. Most sequence differences among mammals are likely to be random variations that preserve the physiological function of PrP (Figure 1B). Yet, some differences should be responsible for the unequal susceptibility to prion diseases, providing additional clues for uncovering the mechanisms underlying PrP dynamics. This is supported by extensive evidence, including the generation of rabbit/mouse chimeric PrP (Vorberg et al., 2003) and expression of PrP in heterologous systems (Vidal et al., 2015, 2020; Bian et al., 2017).

Determination of NMR structures of PrP from dog, horse, rabbit, and pig had the goal of identifying structural features responsible for stabilizing the soluble conformation and inhibit misfolding (Figure 1B) (Lysek et al., 2005; Khan et al., 2010; Perez et al., 2010; Wen et al., 2010). These studies found surprisingly high preservation of the general structure of the globular domain with minor local changes that do not provide a common mechanism to explain conformational stability. The structural changes in rabbit and horse PrP map to the CT3DD, where the β2-α2 loop is stabilized by increased contacts with helix 3 (Lysek et al., 2005; Khan et al., 2010; Perez et al., 2010; Wen et al., 2010). In rabbit PrP, S174 (N174 in most animals) is proposed to participate in a helix-capping domain supported by a double hydrogen bond with N171 (Khan et al., 2010). Horse PrP carries a substitution in S167 (D in most animals) that sits at the center of the critical β2-α2 loop and favors a 3_10_-turn (Perez et al., 2010). PrP from dogs, wolves, and other canids are among the most difficult to convert *in vivo* and *in vitro*, and this resistance is linked to D/E159 (N159 in most animals) (Lysek et al., 2005; Fernandez-Borges et al., 2017; Vidal et al., 2020). This increase in negative surface charge has long-range effects decreasing the population of the short β-sheet (Lysek et al., 2005). Lastly, marsupials as a group have no known prion diseases; the tammar wallaby carries multiple substitutions in the CT3DD, including A225-A226, and shows a 3_10_-turn confirmation for the β2-α2 loop.

The 3_10_-turn received additional attention with the discovery that the transmission of CWD was highly dependent on this domain. CWD prions can be transmitted among cervids (deer, elk, moose, reindeer) but not to other animals, including closely related ungulates like sheep and cattle. This species barrier has been mapped to the different conformations of the β2-α2 loop, which forms a rigid loop in cervids but is flexible in sheep and cattle PrP (Kurt et al., 2009, 2015; Sigurdson et al., 2010; Kurt and Sigurdson, 2016). Deer and elk PrP have two substitutions at 170 (S → N) and 174 (N → T) that favor the rigid loop. Replacement of these two residues impedes transmission to cervid PrP and allow for transmission to other species (Kurt et al., 2014a,b, 2015). However, this species barrier is not only determined by the presence of the 3_10_-turn since a rigid loop supported by a D167S substitution (horse-like) does not support CWD transmission to cervids (Bett et al., 2012). Notably, *in vitro* experiments identified a sheep-specific residue at 208 that also contributes to the sheep-cervid prion barrier (Harrathi et al., 2019). This suggests that additional structural elements contribute to the conformational dynamics of the CT3DD to restrict PrP/PrP interactions that regulate PrP misfolding and templated conversion.

The knowledge of the structural features of PrP and the impact of sequence variations from animals or pathogenic mutants provide an excellent opportunity to understand the correlation between PrP function and dysfunction. These differences have been assayed in many systems *in intro* and *in vivo*. We introduce next the advantages of using transgenic *Drosophila* as an efficient model to examine the consequences of specific mutations *in vivo*.

### Drosophila as a model to dissect animal biology: from genes to behavior

*Drosophila* is tiny fruit fly with a large impact in biomedical research considering that six Nobel Prizes in Medicine and Physiology were awarded to work with this humble fly, three of them in this century. The high accessibility of *Drosophila* to manipulations of its genome along with the improved technologies for identifying molecular, cellular, or behavioral perturbations makes the fly an outstanding complement to study human disease, including cancer, developmental disorders, and neurological and behavioral disorders including neurodegeneration, among others (Ugur et al., 2016; Ma et al., 2022). This is supported by the finding that around 65% of genes involved in human diseases are highly conserved in *Drosophila* (Chien et al., 2002; Yamamoto et al., 2014). Since we are interested in brain disorders, an important aspect of using the fly brain as a model system is the conservation of the brain as a functional organ. The basic function of the nervous system, from ion channels and neurotransmitters to neuron and glia physiology, is highly conserved among animals supporting a common evolutionary origin. With only 10^5^ neurons and 10^7^ synapses, the fly brain does not have by far the capabilities of the exquisite human brain. Yet the range of behaviors of the fruit fly is quite complex for a brain that small (Ugur et al., 2016; Ma et al., 2022). The distinct anatomic organization of the insect and vertebrate brains may argue for different organizing principles and origins. Still, molecular markers reveal a conserved organization along the main axes between the fly and the vertebrate brains, with shared markers for hindbrain, midbrain, and forebrain supporting a shared origin of the brain (Reichert and Simeone, 2001; Hirth, 2010). Despite the differences in brain size and outputs, neuronal complexity is likely encoded and limited by conserved transcription factor as suggested by the finding that the visual system of *Drosophila* and mice each contains around 115 unique neuronal types (Fischbach and Dittrich, 1989; Dacey and Packer, 2003).

### Drosophila models of proteinopathies and prionopathies

A class of brain disorders that have been studied extensively in flies are neurodegenerative diseases, in particular, those caused by progressive protein aggregation (Surguchov, 2021; Nitta and Sugie, 2022; Santarelli et al., 2023; Varte et al., 2023). Between 1998 and 2001 several proteinopathies (three polyglutamines and tau) were modeled in flies for the first time, demonstrating that the proteins responsible for these conditions in humans preserve their toxic properties when expressed in flies (Warrick et al., 1998; Fernandez-Funez et al., 2000; Kazemi-Esfarjani and Benzer, 2000; Wittmann et al., 2001). Many other disease models were subsequently developed to leverage the advanced *Drosophila* genetics to dissect the molecular mechanisms mediating protein aggregation and toxicity (Rincon-Limas et al., 2012; Bolus et al., 2020; Nitta and Sugie, 2022; Pan et al., 2023). Following on our earlier success, we and others modeled relevant aspects of PrP biology in flies, including neurotoxicity, aggregation, and transmission (Fernandez-Funez et al., 2017; Myers et al., 2020; Bujdoso et al., 2022). PrP appeared as a new protein in the chordate linage (Ehsani et al., 2011) and as such it is not conserved in invertebrates, an exception to the high conservation of human genes causing disease. This lack of conservation does not prevent the modeling of prion diseases. In fact, the lack of conservation can be an advantage because it provides a “clean” cellular environment to model PrP misfolding and toxicity. This is in contrast to mammals, which not only express PrP broadly but also have two PrP paralogs, Doppel and Shadoo (Watts and Westaway, 2007). Since the PrP pathology is mediated by age-dependent misfolding and aggregation, replicating these features in flies provides access to investigate the cellular processes regulating misfolding (upstream) and the pathways disrupted by PrP (downstream). The first fly models of prionopathy established that flies replicate key features of prion diseases, including age-dependent neurodegeneration and misfolding into disease-relevant conformations (Gavin et al., 2006; Fernandez-Funez et al., 2009). We and others followed by showing that *Drosophila* expressing PrP-WT from dog, horse, or rabbit display no neurotoxicity and lower aggregation compared to those expressing mouse, hamster, ovine, bovine, or human PrP (Fernandez-Funez et al., 2009, 2010; Thackray et al., 2012b, 2021; Sanchez-Garcia and Fernandez-Funez, 2018; Myers et al., 2022). The preservation of intrinsic properties, including biogenesis, folding, and age-dependent misfolding and toxicity, demonstrates that flies provide an appropriate cellular environment for expressing mammalian PrP. Moreover, transmissible models of prion disease with ovine and bovine PrP have been stablished as potential platforms for bioassays and for the discovery of the mechanisms mediating the templated conversion of PrP (Thackray et al., 2012a, 2014, 2016, 2018, 2021; Bujdoso et al., 2015).

Our recent efforts have centered on the generation of *Drosophila* models with robust phenotypes that can be used as platforms for genetic screens and for identifying the intrinsic determinants of PrP toxicity. We hypothesized that human PrP would be more toxic than rodent PrPs due to the heterogeneity of prion diseases in humans, suggesting the natural formation of conformational several strains. We generated transgenic models carrying human and rodent PrP constructs and showed that human PrP is qualitatively more toxic than mouse or hamster PrP (Bischof et al., 2007). Human PrP is so far the only PrP model with a robust eye phenotype (Fernandez-Funez et al., 2017; Myers et al., 2022), which is critical for performing fast phenotypic screens and has played a critical role in many *Drosophila* models of neurodegeneration (Jackson et al., 1998; Warrick et al., 1998; Fernandez-Funez et al., 2000; Kazemi-Esfarjani and Benzer, 2000; Wittmann et al., 2001; Jackson et al., 2002; Crowther et al., 2005; Ritson et al., 2010; Casas-Tinto et al., 2011; Mizielinska et al., 2014). It is noteworthy that human PrP-WT is highly toxic when expressed in fly neurons. This should not be completely surprising since many brain proteinopathies are sporadic (e.g., Alzheimer’s, Parkinson’s disease), meaning that a WT protein misfolds and aggregates in the absence of mutations or exogenous seeding agents. This strange quality is due to the intrinsic properties of amyloid proteins, which are characterized by the presence of prion-like, low complexity domains highly prone to misfold (Daskalov et al., 2021; Gil-Garcia et al., 2021; Sprunger and Jackrel, 2021). Sporadic prion diseases are by far the most common form of these diseases in humans despite the attention placed on its transmissible forms due to their novelty and public health relevance. Sporadic prion diseases manifest around the 5th decade in humans but are rare in rodent models, mostly due to high expression (Westaway et al., 1994; Huang et al., 2007; Chiesa et al., 2008). The fast misfolding and toxicity in flies can be explained by a shift in conformational dynamics due to relative high expression and the hijacking of the biogenies and secretion cellular machinery due to the lack of endogenous PrP. It is important to remember that high expression of PrP from rabbit, dog, or horse PrP are not toxic in flies, whereas rodent PrP cause weaker phenotypes than human PrP (Fernandez-Funez et al., 2010; Sanchez-Garcia and Fernandez-Funez, 2018; Myers et al., 2022). Thus, the high toxicity of human PrP must be encoded in its subtle sequence differences with animal PrPs.

We have leveraged the human PrP model for the fast analysis of substitutions on human PrP that lower toxicity. Once the constructs are introduced in flies, a first generation cross can tell us in 10 days whether a mutation has any impact on the eye in living flies under the stereoscope. We recently introduced several candidate residues expected to lower PrP toxicity with unequal results (Myers et al., 2022, 2023). We examined the functional impact of introducing N/D159, D/S167, and N/S174 in the context of dog, horse, rabbit, mouse, or human PrP in transgenic flies. Expression of mouse PrP-N159D in flies showed less toxicity and lower accumulation of pathogenic PrP conformations than those expressing mouse PrP-WT, lending support for the protective role of D159 (Sanchez-Garcia et al., 2016). Flies expressing dog PrP-D159N or horse PrP-S167D displayed robust toxicity in the mushroom body assay vs. their non-toxic WT versions (Sanchez-Garcia and Fernandez-Funez, 2018), also supporting their importance for the conformational stability of dog and horse PrP. In contrast, rabbit PrP-S174N had no effect on flies. Unexpectedly, expression of human PrP carrying the reciprocal substitutions (N159D, D167S, and N174S) showed little protective effect in fly toxicity, if any, indicating that these residues do not confer conformational stability of human PrP *on their own* (Myers et al., 2022, 2023). Considering the inconsistent effects of these substitutions and their dependence on the PrP backbone, the role of D159, S167, and S174 in the dynamics of the CT3DD is unclear. In the case of S174, the proposed helix-capping domain may be relevant only in the X-ray structure (Lysek et al., 2005; Khan et al., 2010), suggesting that unknown residues govern rabbit PrP stability. The weak impact of these substitutions made us develop a new strategy to extract critical knowledge from the PrP structure to inform the generation of PrP substitutions that more effectively inhibit human PrP toxicity. Currently, it is unclear how the CT3DD is stabilized in these animals, which likely involves multiple residues with secondary but cooperative roles. These are likely to be conservative substitutions that have, so far, escaped scrutiny. Other considerations beyond fixed conformations are at play, and we propose that conformational dynamics holds the mechanistic clues that encode for toxicity of transmissibility.

### Conformational dynamics of PrP by molecular modeling

Building on the abundance of experimental structures discussed above, the globular domain of PrP has been the subject of extensive computational studies starting in the early 2000’s. Molecular dynamics (MD) simulations provide access to the complex internal motions of atoms within proteins, which provides information about different conformational states, including protein folding and misfolding (Karplus and McCammon, 2002; Karplus and Kuriyan, 2005; Chiti and Dobson, 2006; Schaeffer et al., 2008; Glazer et al., 2009; van der Kamp and Daggett, 2011). With the increase in computational power and advances in simulation methods, this technique can deliver fundamental insights into protein dynamics and folding. MD simulations were first applied on PrP to study basic conformational dynamics of rodent and human PrP and the impact of pathogenic mutations (Zuegg and Gready, 1999; Guilbert et al., 2000; Parchment and Essex, 2000; El-Bastawissy et al., 2001). One of the earliest studies showed that at low pH, the β-sheet extends to include the N-terminus and almost the entire β2-α2 loop (Alonso et al., 2001). These data were used to build a protofibril model in which the extended β-sheet served as the interface for the stacking of PrP monomers (DeMarco and Daggett, 2004). A work combining a structural motif database search and MD simulations identified two main regions of instability in the globular domain: distal helix 2 and distal helix 3 (Dima and Thirumalai, 2004). Recent studies identified the N-terminus, β-sheet, β2-α2 loop, and the C-terminus of helix 3 as the PrP domains most prone to unfolding (Chamachi and Chakrabarty, 2017; Singh et al., 2017). Studies focused on the CT3DD uncovered a key role for the solvent exposure of the highly conserved Y169 in stabilizing the 3_10_-turn (Huang and Caflisch, 2015a,b; Caldarulo et al., 2017). Lastly, the systematic characterization of the secondary structures and flexibility for many PrP species identified a critical salt bridge between R164 and D178 for the β2-α2 loop stability (Zhang and Wang, 2016; Zhang, 2018).

**Figure 2:**
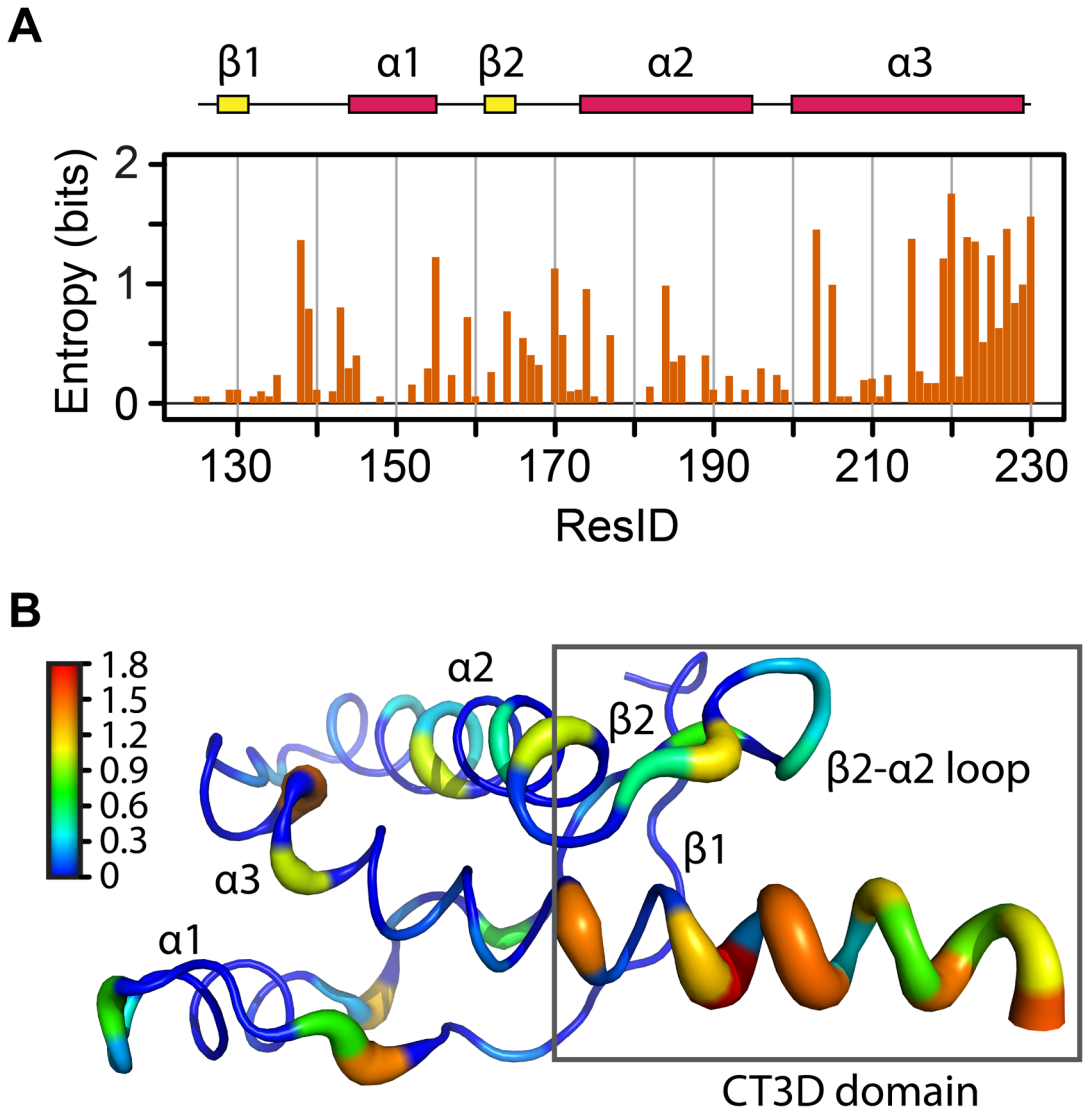
Sequence entropy in the PrP globular domain. **(A)** The sequence entropy for the PrP structured domain is plotted as a function of the residue number in units of bits. **(B)** The sequence entropy is mapped onto the human PrP structure, with thicker ribbons corresponding to higher entropy as shown by the color bar.

Numerous pathogenic mutants have been probed by MD simulations, with T183A showing the strongest destabilization. These mutations cause global effects on PrP folding due to the disruption of key stabilizing interactions (Rossetti et al., 2010, 2011; Sanz-Hernandez et al., 2021). In general, these mutations promote misfolding by first disrupting the interaction between the β-sheet and helix 1 and the region composed of the helices 2 and 3 (Liemann and Glockshuber, 1999; Vanik and Surewicz, 2002; Apetri et al., 2004, 2005). These domains are normally stabilized by four “gatekeeper” interactions (D178–R164, T183–Y162, H187–R156, and D202–R156), each containing a pathogenic mutation (underlined) (Hadzi et al., 2015; Palaniappan et al., 2021). The second step involves the increased surface exposure of the β2–α2 loop, mainly mediated by Y169, and disrupted interactions within the CT3DD. The E219K protective polymorphism exhibits stronger aromatic and hydrophobic interactions by M166, F175, Y218, and Y225, all within the CT3DD, which favors the 3_10_-turn. Overall, it appears that the CT3DD shows low tolerance for pathogenic mutations that can result in catastrophic effects on PrP biogenesis (Figure 2) but can tolerate a protective mutation. These interesting questions can be investigated by both computational and functional studies.

### Sequence entropy

A classic NMR/modeling study mapped the variability of 23 sequences to identify relevant functional subdomains within the PrP globular domain (Billeter et al., 1997). To gain further insight into how sequence variation is distributed across this domain, we performed sequence alignment of 156 PrP sequences and used the sequence entropy as a reporter for variability (Figure 2A). The region spanning L125-S230 contains 37 amino acids with zero entropy (no sequence variation), suggesting that these residues are likely essential for PrP folding and / or function, like C179 and C214, which form a critical disulfide bridge between helices 2 and 3. These highly conserved residues are mainly located on the helices, except on the C-terminal of helix 3 (Figure 2B). Another position with zero entropy is Y169, which forms interactions that control the loop dynamics, in which the 3_10_-turn is in slow NMR exchange (in the order of milliseconds) with a β-turn (Damberger et al., 2011; Caldarulo et al., 2017). In mouse PrP, the Y169G/A substitutions stabilize the β-turn, demonstrating the importance of Y169 in the dynamics of the entire CT3DD (Damberger et al., 2011).

**Figure 3:**
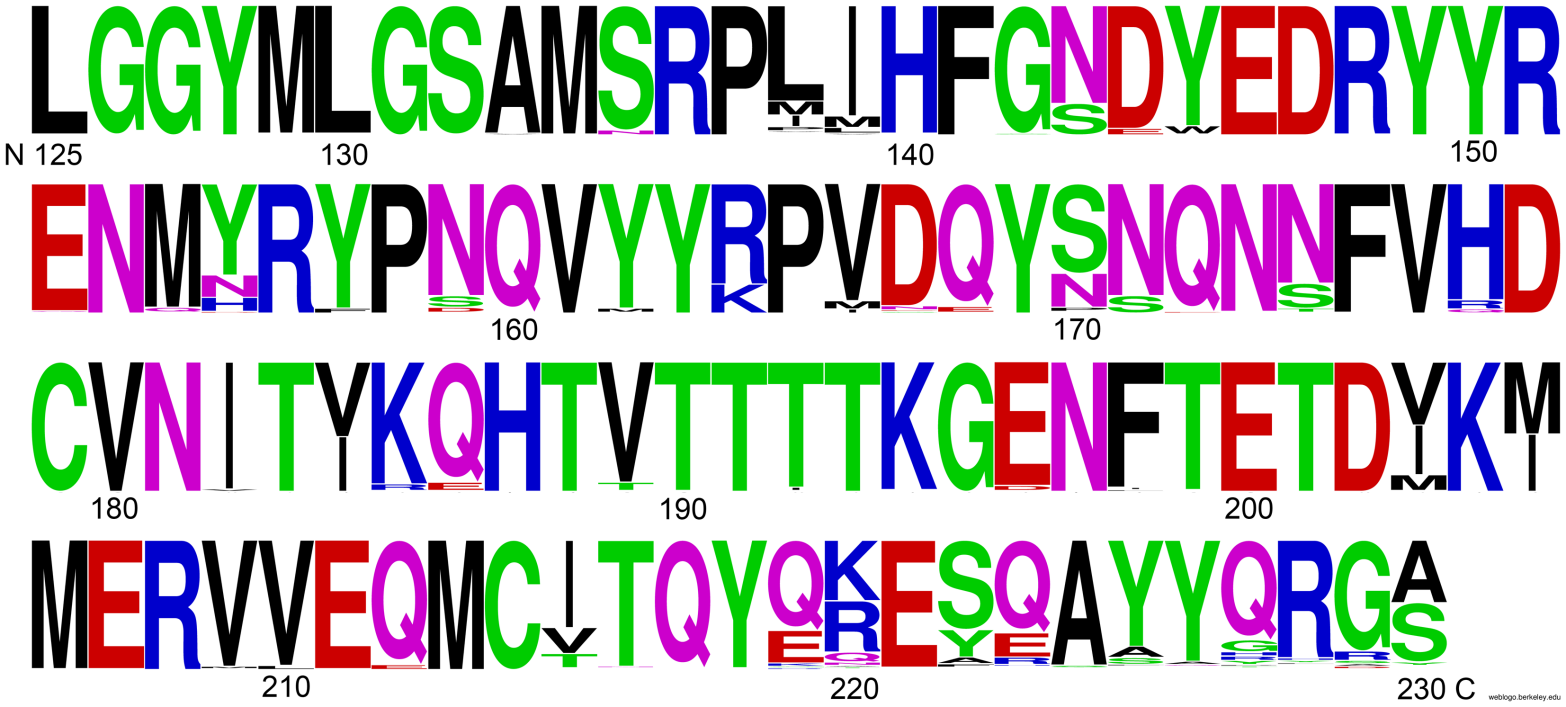
Amino acid prevalence in the globular domain. Amino acids are colored by their properties, and sizes correspond to their relative frequency for a given position. Generated with WebLogo.

The residues with larger entropy are defined as those with values over 0.80 bits. This threshold was chosen by calculating the mean entropy and the standard deviation for all residues plus one standard deviation to the mean. 17 amino acids showed large entropy, 12 of them located in the CT3DD, but most are conservative changes (Figure 3). Positions 138, 184, 203, and 205 are conserved substitutions that involve the hydrophobic scaffolding of helices 2 and 3. In the β2–α2 loop, conservative substitutions include R/K164, M/V/I166, D/N/S167, Q/E168, S/N170, S/N171, and S/N174. Notably, human PrP is one of the few mammals that carries M166 and E168. Lastly, the C-terminus of helix 3 reveals many conservative substitutions (e.g., Q/E219 and 223, K/R/Q220, A/S230) (Figure 3), yet Y/A at 225/226 appear as significant substitutions.

These findings identify the CT3DD as a region with large sequence entropy. High tolerance for conservative mutations with no effect on PrP function would avoid selective pressure and create the potential to impart more nuanced characteristics, such as changes in flexibility and dynamics that may contribute to the defense against zoonotic prion transmission. Carnivores in the canid family and omnivores like humans may have acquired changes in this domain to prevent disease transmission from the consumption of prion-contaminated meat (Fernandez-Borges et al., 2017; Vidal et al., 2020). Given the environmental stability of prions, herbivores like horses and rabbits may have also acquired protective substitutions for the same reason. This argument is mostly speculative because other carnivores like felines and mustelids are vulnerable to prion transmission as are many herbivores that share their environment with deer, moose, sheep, and goats.

### Backbone conformational flexibility

**Figure 4:**
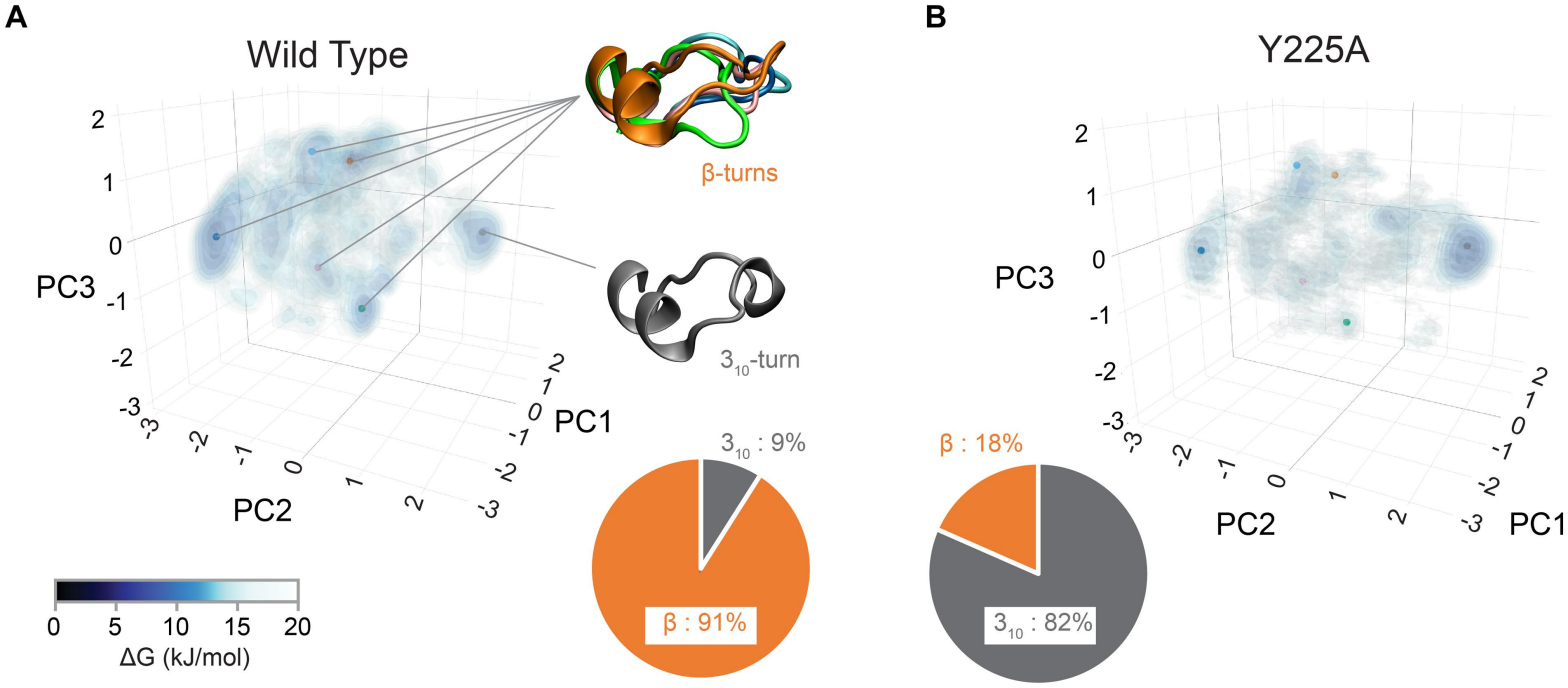
Structural diversity of the CT3DD in human PrP. Structural information for the β2-α2 loop from MD simulations (Myers et al., 2023) was used to created Gibbs free energy isocontours in the space described by the first three principal components calculated using the 𝜑/𝜓 dihedrals. Darker colors correspond to stable regions (see color bar). The pie-chart shows the relative ratio of 3_10_- and β-turn conformations. **(A)** Human wild-type simulations. The points indicate the center of each of the clusters describing the six main different conformations that were identified. Representative structures of the five β-turns conformations as well as of the 3_10_-turn conformation are shown. **(B)** The data from human PrP-Y225A MD simulations are plotted using the same approach as above.

**Figure 5:**
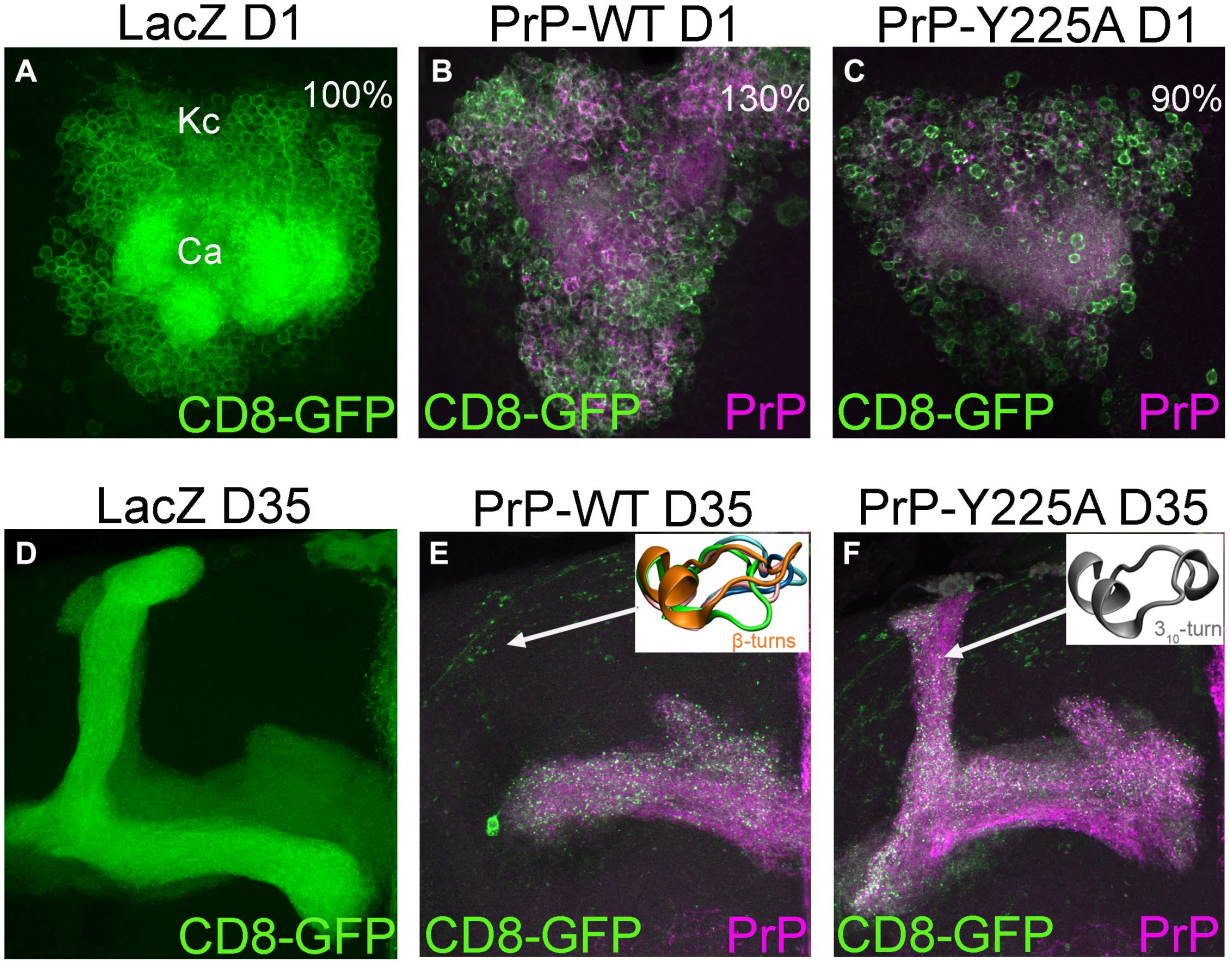
Degeneration of mushroom body neurons in *Drosophila*. **(A,D)** Control flies expressing the reporter LacZ in mushroom body neurons visualized with CD8-GFP (UAS-LacZ/UAS-CD8-GFP; OK107-Gal4). These clusters contain about 2,000 neurons [**(A)** The Kenyon cells, Kc] tightly packed in the posterior brain. Their dendritic fields or calyces (Ca) are located underneath the cell bodies. These neurons project to the anterior brain and project into dorsal or medial projections **(D)**. **(B,C)** 1-day-old flies expressing human PrP-WT (UAS-human PrP-WT/UAS-CD8-GFP; OK107-Gal4) show expansion of the Kc clusters, averaging 30% increased surface compared to controls, whereas flies expressing Y225A (UAS-human PrP-Y225A/UAS-CD8-GFP; OK107-Gal4) show slightly smaller area. **(E,F)** 35-day-old flies expressing PrP-WT show progressive loss of axonal projections and this loss is minimized in flies expressing Y225A. Representative loop conformations are shown as insets. See Myers et al. (2023) for additional details.

In our recent work, we identified the β2-α2 loop in human PrP as a region of high conformational polymorphism (Myers et al., 2023). Using the detailed data from enhanced sampling MD simulations, we characterized the conformational landscape of the β2-α2 loop by examining the φ/ψ dihedral angles defining its backbone secondary structure (Huang and Caflisch, 2015a,b). Using principal component analysis, we plotted the Gibbs free energy along the top three vectors and identified several energy minima corresponding to five distinct β-turns (91% of the population) and a 3_10_-turn (9%) (Figure 4; Myers et al., 2023). These findings describe a highly dynamic β2-α2 loop in human PrP-WT that can explore multiple conformations separated by low energy barriers, each stabilized by different networks of intramolecular interactions (Myers et al., 2023). Next, we introduced a single amino acid substitution from rabbit PrP (also in pig and wallaby) at the end of helix 3 (human PrP-Y225A) and conducted similar analyses. Y225A severely lowered the conformational dynamics of the β2-α2 loop and showed a significant preference for the 3_10_-turn, which is now populated in 82% of the samples, leaving only 18% for the β-turns (Figure 4). This drop in dynamics was accompanied by a reduced overall hydrophobic exposure (due mainly to Y169), suggesting that the 3_10_-turn provides a more stable conformation less likely to misfold. We next asked if this shift toward the 3_10_-turn had an impact on the toxicity and aggregation of human PrP-Y225A. We created transgenic flies expressing human PrP-WT and Y225A in highly comparable conditions. Y225A was less toxic than WT in two assays in flies, the eye and the mushroom bodies. Observing the suppression of toxicity in the eyes gave us the confidence to examine brain neurons, which require aging the flies, brain dissection, and imaging of whole-mount brains by confocal microscopy. This assay revealed a new phenotype for human PrP-WT in mushroom body neurons resulting in the expansion of the clusters containing the cell bodies, a phenotype not found in flies expressing Y225A (Figures 5A–C; Myers et al., 2023). In flies aged for 35 days, flies expressing PrP-WT show significantly more degeneration of mushroom body neurons than those expressing Y225A (Figures 5D–F). Lastly, we examined the aggregation of human PrP in flies by taking advantage of precipitation with NaPTA, a compound that specifically binds misfolded PrP. As expected, Y225A showed lower propensity to aggregate than WT, which is consistent with the lower toxicity and the increased conformational stability in MD simulations (Myers et al., 2023). These assays allowed us to correlate high dynamics of the loop and the population of the β-turns in PrP-WT with high toxicity in fly brains, whereas the high stability of the 3_10_-turn correlates with the lower toxicity of Y225A. These results also illustrated the important role of helix 3 in modulating loop dynamics. Yet, since Y225A still shows significant remaining toxicity, there must be a significant contribution to PrP misfolding and toxicity from other residues. Overall, we postulate that the enhanced conformational polymorphism of the β2-α2 loop may be a marker for the propensity of human PrP to spontaneously unfold and aggregate, opening new avenues to test this hypothesis in future studies.

**Figure 6:**
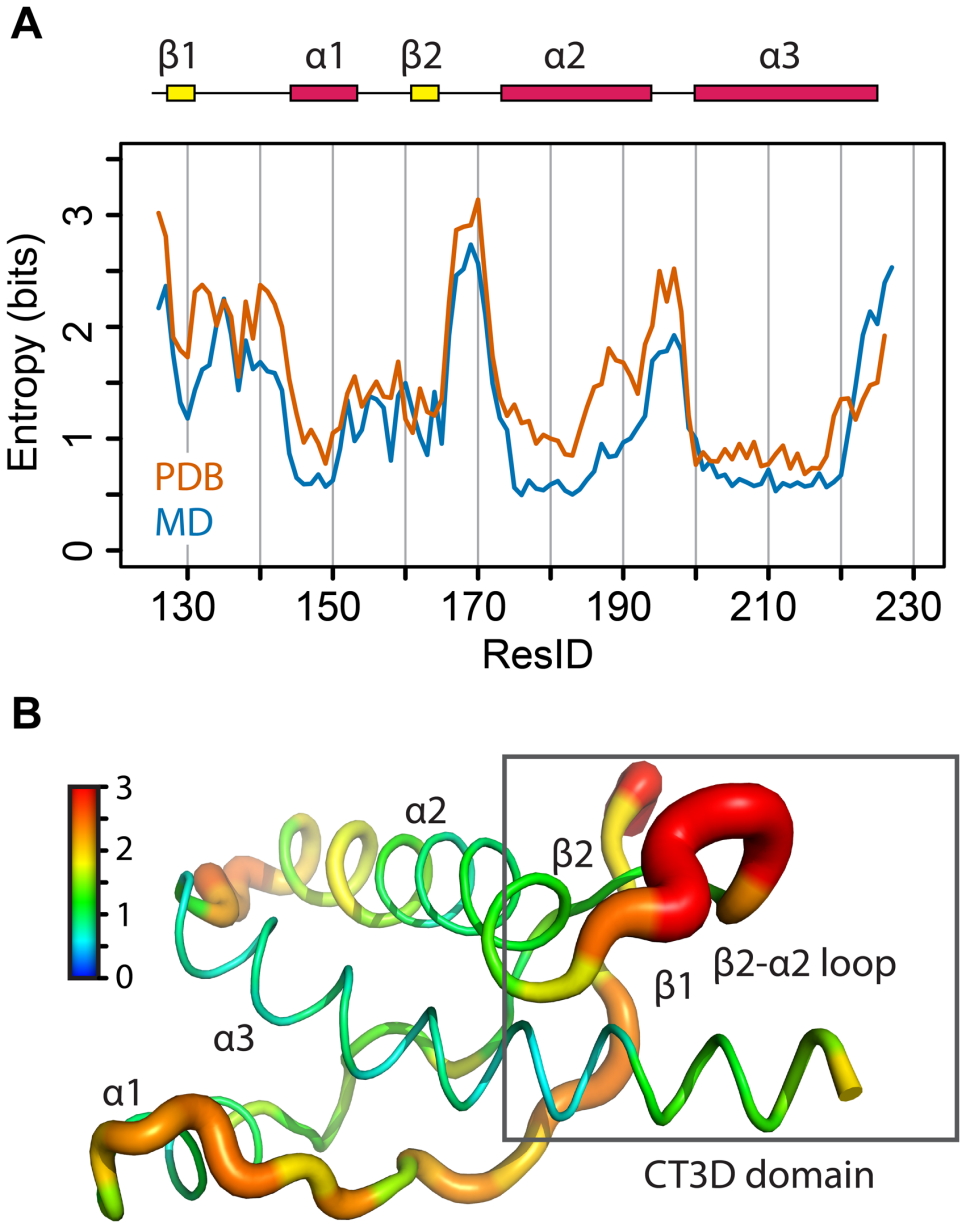
Structural entropy in the globular domain of PrP. **(A)** The φ/ψ dihedral entropy for the PrP structured domain is plotted in orange as a function of the residue number in units of bits. In blue, the 𝜑/𝜓 dihedral entropy from MD simulations of human PrP is plotted. **(B)** The φ/ψ dihedral entropy from the PDB data is mapped onto the human PrP structure with thicker ribbons corresponding to higher entropy as shown by the color bar.

To further expand on this idea, we mapped the conformational flexibility of the entire backbone of the globular domain. To this end, we calculated the φ/ψ dihedral angles for available PrP structures from PDB data and calculated the entropy for each angle (Figure 6A). The maximum dihedral entropy for a random angle distribution is 3.6 bits. The conformational entropy plot shows that the β2-α2 loop has the largest entropy of the globular domain, with a peak of over 3 bits, close to a random angle distribution (Figure 6A). Two other loops also show high entropy, the β1-α1 loop and the α2-α3 loop, yet the β2-α2 loop has the highest entropy despite the β1-α1 loop being longer and similarly exposed to the solvent. We next overlapped the results from 840 mammalian PrP PDB structures (in orange) with our MD simulation (in blue) (Figure 6B). Both sets are remarkably similar and identify the β2-α2 loop as the region with the highest conformational entropy suggesting a high sensitivity to changes in its immediate surroundings, namely the CT3DD. Taken together, these observations are compatible with the description of the β2-α2 loop as the “weak link” in the conformational stability of PrP, while the C-terminus of helix 3 contributes to (de)stabilizing the loop through subtle sequence variations.

### Prp as a model to uncover the principles governing sequence→function

So far, the PrP community has identified several regions of interest likely to modulate PrP misfolding and disease states. Yet the traditional approach to link protein sequence and function – sequence → structure → function – has produced limited advances in uncovering the mechanisms governing PrP misfolding. We propose a paradigm shift to study PrP function that goes beyond static domain structure(s) and, critically, incorporates the dynamics of its key subdomains: sequence→ structure → *dynamics* → function. PrP is an excellent model for these studies because a wealth of structural data is available in the form of NMR and X-ray structures that can be leveraged to answer these important questions. These structures can be used in computational studies that allow for a broader exploration of the conformational landscape, which describe a dynamic state that more closely resembles the behavior of proteins at atomic level. Additionally, this approach can benefit the study of other amyloidogenic proteins causative of proteinopathies, some of them highly prevalent. Amyloids contain low complexity or partially disordered domains likely to exhibit high conformational dynamics. These dynamic states shift the conversation from static local conformations to transitions (dynamics) between multiple states. From here, the mechanisms stabilizing each state, energy barriers, relative populations of different states, and transitions between states can be analyzed. These analyses add significant complexity to the study of PrP and proteins with prion-like domain, but they open a window into the intramolecular interactions and energies that govern protein behaviors.

**Figure 7:**
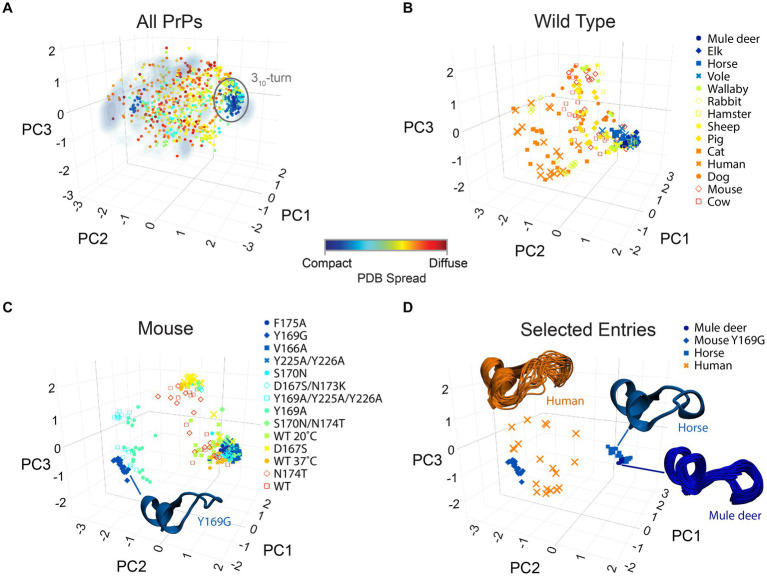
Structural diversity of PrP. The structures of the β2-α2 loop from all PrPs available in PBD are mapped onto the axes obtained from principal component analysis of the φ/ψ dihedrals. The markers describe the spread within each PDB set (usually 20 models) as described by the color bar. **(A)** All 840 PrP structures in PDB are mapped onto the axes obtained from MD simulations for human PrP as reference (Figure 4A; Myers et al., 2023). **(B)** The 280 PrP-WT structures available in PDB are shown. **(C)** The 280 mouse PrP (WT and mutant) structures available in PDB are reported. The structure of the β2-α2 loop for Y169G is shown as a cartoon. **(D)** The complete ensemble of 20 PDB structures for selected PrPs are shown to illustrate the population of specific conformations (mule deer, horse, mouse Y169G) or their conformational diversity (human).

PrP’s CT3DD and its β2-α2 loop are particularly interesting for their high sequence and conformational entropy (Myers et al., 2023). The logical next steps are to develop models that explain how this subdomain is (de)stabilized followed by experimental manipulations of key residues that examine their impact on the local and global PrP dynamics and their *in vivo* properties, like toxicity. As a first step toward relating PrP toxicity to its dynamics, we analyzed the conformation of the β2–α2 loop on 42 different PDB models of PrP, each containing around 20 structures for a total of 840 structures. Using the same approach that we used for MD simulations of human PrP, we identified three vectors that described combinations of φ/ψ dihedral angles for the β2–α2 loop that result in the greatest discrimination in loop conformations (Figure 7A) (see Methods, Bujdoso et al., 2022). Because a PDB for an NMR structure is deposited as an ensemble of 20 models, we calculated the conformational variability within each PDB ensemble as the standard deviation in the distance between the models. This is represented as a color-coded PDB Spread for the diversity of conformations of the β2–α2 loop, where each spot represents an individual PDB model (Figure 7A). The 840 PDB models were then projected onto the Gibbs energy density profile obtained from the human PrP MD simulations as reference, showing good overlap (Figure 7A). This observation strengthens the claim that MD simulations offer a valid and accessible method to study PrP conformational dynamics. The *Compact PDBs* (low spread) cluster around the 3_10_-turn region except the anti-3_10_-turn mutation Y169G, reinforcing the proposed stability imparted by the 3_10_-turn. Limiting the analysis to only WT models, they split into low spread and presence of the 3_10_-turn (deer, elk, horse, and vole) and high spread and population of β-turns (cat, human, dog, mouse, and cow) (Figure 7B). Because mouse PrP has been the subject of extensive structural studies, it provides a unique opportunity to evaluate the effect of single substitutions on the structure and dynamics of the β2–α2 loop (Figure 7C). Mouse PrP-WT shows high dynamics that samples β-turns and the 3_10_-turn (Figure 7C). Yet, F175A, V166A, Y225A-Y226A, and S170N preference for the 3_10_-turn. In contrast, Y169G behaves as an anti-3_10_-turn that, surprisingly is highly stable and favors one type of β-turn (Figure 7C; Damberger et al., 2011). Thus, stable conformations other than the 3_10_-turn are possible and could be exploited for therapeutic approaches aiming at limiting the dynamics in this region. Lastly, we highlight representative examples of conformational diversity, like the mule deer PrP, which has the most compact ensemble appreciated from its largely overlapping structures (Figure 7D). This is in stark contrast with human PrP, which shows widespread distribution. Horse PrP-WT and mouse PrP-Y169G both show low dynamics but prefer different conformations, 3_10_-turn for horse PrP and β-turn for mouse PrP-Y169G.

Overall, this analysis reveals that the rich information present in the PDBs is sufficient to begin exploring PrP conformational dynamics. Yet, NMR is not a high yield experimental tool for expanding our understanding of the consequences of introducing additional mutants, particularly in human PrP. We also need to consider that the variability within a PDB ensemble may be biased by the NMR refining restraints. To systematically study the effects of substitutions on the conformational dynamics of the β2–α2 loop is therefore important to employ methods that allow for standardized and reproducible approaches, which is provided by the combination of MD simulations and *Drosophila* functional experiments that we have developed. Our recent work identified residues within the CT3DD contributing to the stability of the 3_10_-turn, providing testable hypotheses. Based on our recent experience, single substitutions in the human PrP backbone are likely to have limited impact on its dynamics because of the coordinated work of several residues. Thus, we have started to examine the impact of single and double substitutions to determine their cumulative effects in MD simulations and flies. We are considering triple mutants to address the significant contribution of conserved residues in helix 3 to the stability of the CT3DD. The MD simulations conducted so far contain rich information that we can exploit as our working hypotheses are refined over time. We can also add metadynamics (Laio and Parrinello, 2002) to our computational work to study in more detail the transition between relevant states: the 3_10_-turn and a common β-turn. Having the ability to correlate the computational data with *in vivo* data in a timely manner is highly valuable because it allows us to feed that information back into our working models to identify the most relevant combinations of residues modulating the CT3DD. We believe this approach can provide sufficient resolution to develop models that can be replicated in mammalian cellular models and, eventually, tested in transgenic mice.

### Concluding remarks

We face parallel challenges in dissecting the rules governing PrP misfolding and toxicity that inhabit different spheres of knowledge. The first challenge is to develop high resolution models for the intrinsic dynamics of the CT3DD that can accurately predict the consequences of disrupting this region. The second challenge is to efficiently produce evidence in living systems for the functional impact of the same perturbations in relevant toxicity and aggregation assays. Generating functional evidence in living systems is critical but it is time consuming and expensive, even when using simplified models like *Drosophila*, cell culture, and *in vitro* systems. Thus, detailed guidance from PrP structure AND dynamics are critical to predict which residues to prioritize. Our approach combining MD simulations with *Drosophila* expressing PrP allows for efficient and economic functional tests of candidate residues before launching into more expensive and time-consuming experiments in mice. Our approach models spontaneous PrP misfolding, which accounts for the sporadic etiology of prion diseases in human. This may be an advantage compared to mouse models, which only show sporadic disease manifestations under high overexpression conditions (Westaway et al., 1994; Huang et al., 2007; Chiesa et al., 2008). Working with flies has drawbacks as well, including the smaller amounts of PrP recovered for biochemistry or transmission experiments and the lack of spontaneous production of protease resistant PrP, indicating the absence of relevant cofactors or incubation time. The output guiding our experiments is eliminating the spontaneous toxicity of human PrP in transgenic flies. This objective aligns better with the goal of finding a cure for these devastating diseases than increasing PrP toxicity or promoting its transmissibility. Eventually, this knowledge can be leveraged to dissect the rules governing the misfolding and aggregation of other amyloids, which has a much larger potential for impact on public health due to the high prevalence of several proteinopathies among the elderly.

## Materials and methods

Analyses were performed using R v. 4.0.4, and sequences were aligned with the bio3D package v. 2.4–4 interfaced with Muscle v. 3.8.31.

### Sequence analysis

PrP sequences were identified by performing a BLAST search of the TrEMBL and Swiss-Prot databases against the human prion protein sequence from L125 to S230. Hits with less than 70% identity were removed. Of the remaining 193 sequences, we discarded a few that missed large sections (10 residues or more) when aligned to the human sequence, as well as any duplicate sequence from the two databases. The resulting set contained 156 sequences. Following sequence alignment, only the columns corresponding to the range L125-S230 of the human sequence were retained. The Shannon entropy of the resulting 106 sequence positions was calculated for both Extended and Reduced sets. The entropy was mapped onto the PrP structure (PDB ID:1QM1, with manually added G229 and S230 in α-helix conformation) using PyMOL v. 2.3.0. A sequence logo showing the relative prevalence of amino acids was generated on the aligned sequences using the online WebLogo service (version 2.8.2) (Crooks et al., 2004).

### Backbone φ/ψ angles entropy

A database of 840 PrP structures was built using 42 PrP entries from the Protein Data Bank determined by biomolecular NMR (all PDB IDs are provided in the Supporting Information). The *φ/ψ* dihedral angles for the range corresponding to G126-Y226 were calculated with bio3D package – notice that the shortened range used here is due to the lack of structural data for residues outside this range. To calculate the dihedral entropy, the R package “infotheo” (v. 1.2.0.1) was used. First the dihedral angles were discretized using the “Global Equal Width” method into 12 bins, then the entropy for each *φ/ψ* dihedral angle was calculated. Notice that the calculated entropy depends on the number of bins chosen in the discretization step, with more bins resulting in – up to a point – higher *absolute* entropy. Nevertheless, *relative* entropies should be conserved for reasonable choices of the number of bins. As a reference, using our 12 bins selection, the maximum entropy value corresponding to a random distribution is 3.6 bits. Finally, the average of the *φ/ψ* dihedral angles entropy for each residue was used as a measure of the conformational flexibility. The entropy was mapped onto the PrP structure using the same approach described for the Sequence Analysis.

Using the same approach as above, the same quantity was calculated using 40,000 structures of human PrP from molecular dynamics simulations as input. Details of the simulations setup are described in Myers et al. (2023), but briefly these data were obtained from 200 ns of temperature replica-exchange molecular dynamics simulations which efficiently sampled the conformational space accessible to the protein.

### PDB structures conformational variability

To characterize the structural features of the β2–α2 loop across all PrP NMR structures in the PDB, principal component analysis (PCA) of the loop’s backbone dihedral angles was performed with a similar approach as in Myers et al. (2023). Briefly, the sine and cosine of the φ/ψ dihedral angles for the β2–α2 loop (residues 164–175) were calculated for the 840 PDB structures, and then principal component analysis was performed in the sine/cosine space. Then, for every PDB structure, the sine and cosines for the loop’s residues were projected onto the first three eigenvectors obtained from the PCA analysis, resulting in a point in space that describes a particular conformation. Points that are closer in space represent similar structures, and we measured the similarity of the structures in a PDB ensemble (usually 20 structures) as the standard deviation in the distance between the points in the ensemble, which we color-coded as “PDB Spread” in Figure 2.

## Author contributions

All authors listed have made a substantial, direct, and intellectual contribution to the work and approved it for publication.

## Funding

This work was supported by the NIH grant 7R21NS096627-02 and the Winston and Maxine Wallin Neuroscience Discovery Fund award CON000000083928 to PF-F.

## Conflict of interest

The authors declare that the research was conducted in the absence of any commercial or financial relationships that could be construed as a potential conflict of interest.

## Publisher’s note

All claims expressed in this article are solely those of the authors and do not necessarily represent those of their affiliated organizations, or those of the publisher, the editors and the reviewers. Any product that may be evaluated in this article, or claim that may be made by its manufacturer, is not guaranteed or endorsed by the publisher.
